# High-Dose Aluminium Chloride Exposure Disrupts the Renal Cortical Injury–Repair Balance in Rats: Partial Modulation by L-Carnitine Pretreatment

**DOI:** 10.3390/ani16121896

**Published:** 2026-06-18

**Authors:** Faten S. Abo-Zeid, Wiame W. M. Emam

**Affiliations:** Zoology Department, Faculty of Science, Ain Shams University, Cairo 11566, Egypt; faten.sabra@yahoo.com

**Keywords:** kidney, cleaved caspase-3, Ki-67, apoptosis, proliferation, immunohistochemistry

## Abstract

Severe subacute exposure to aluminium chloride (AlCl_3_) impairs renal function and induces cortical tubular injury; however, the concomitant balance between injury and repair in tubular epithelia remains incompletely defined. Accordingly, we aimed to use a high-dose regimen of AlCl_3_ (100 mg·kg^−1^·day^−1^ for 30 days, oral gavage) as a standardised renal stressor in male Wistar rats to quantify shifts along the injury–repair balance in the renal cortex and to test whether L-carnitine (LC) pretreatment (200 mg·kg^−1^·day^−1^) can attenuate these shifts. Twenty rats were assigned to four groups: control, LC alone, AlCl_3_ alone, and LC followed 60 min later by AlCl_3_. On day 31, we assessed body-weight gain, renal functional markers, blinded cortical lesion scoring, quantitative histochemistry, and immunohistochemical profiling of cleaved caspase-3 (apoptotic signalling) and Ki-67 (proliferative engagement) within the same cortical compartment. AlCl_3_ exposure produced a severe renal stress phenotype compared with controls, reducing body-weight gain from 99.8 ± 8.6 to 24.0 ± 8.3 g and increasing serum urea and creatinine from 26.40 ± 3.21 to 48.60 ± 5.81 mg/dL and from 0.606 ± 0.063 to 0.956 ± 0.147 mg/dL, respectively. Cortical injury increased from 0 (0–0) in controls to 15 (15–15) after AlCl_3_ exposure. AlCl_3_ also reduced strong PAS area from 97.92 ± 1.10% to 52.37 ± 14.68% and protein optical density from 0.353 ± 0.020 to 0.269 ± 0.039, while increasing collagen area fraction from 6.92 ± 1.67% to 18.40 ± 3.02% and cleaved caspase-3 from 1.0 (1.0–2.0) to 12.0 (12.0–12.0). Ki-67 labelling declined from 17.80 ± 3.35% to 6.00 ± 1.58%, indicating suppressed proliferative engagement. Compared with AlCl_3_ alone, LC pretreatment showed partial protection, with higher body-weight gain (70.0 ± 15.6 g), lower serum urea and creatinine (21.40 ± 2.30 mg/dL and 0.580 ± 0.084 mg/dL), lower cortical injury burden [3 (3–4)], greater strong PAS area (89.25 ± 2.67%), higher protein optical density (0.354 ± 0.012), lower collagen area fraction (12.26 ± 1.70%), lower cleaved caspase-3 [4.0 (4.0–6.0)], and higher Ki-67 labelling (10.60 ± 2.30%). Residual cortical injury, persistent collagen elevation, and incomplete Ki-67 preservation indicate that LC pretreatment attenuated, but did not fully prevent, AlCl_3_-induced renal cortical alterations. Overall, high-burden AlCl_3_ exposure not only enhanced cell loss but also impaired regenerative renewal, whereas LC pretreatment partially preserved this injury–repair balance.

## Simple Summary

Although high exposure to aluminium is known to damage the kidneys, it remains unclear whether it also weakens the kidney’s ability to repair itself after injury. This study addressed that problem by examining the balance between kidney damage and cell renewal in male rats exposed to a high dose of aluminium chloride. It also tested whether L-carnitine, a natural substance that helps cells produce energy, could support this balance when given before aluminium exposure. Rats were divided into four groups to compare normal kidneys: L-carnitine alone, aluminium chloride alone, and L-carnitine given before aluminium chloride. Aluminium chloride reduced body-weight gain, disturbed blood measures of kidney function, damaged the small kidney tubules that help process fluid into urine, increased scar-like tissue changes, increased signs of cell loss, and reduced signs of cell renewal. On the other hand, L-carnitine pretreatment partly protected the kidney by preserving tissue structure, limiting scar-like changes, reducing several signs of injury, and supporting cell renewal. The main value of this study is that it shows aluminium stress not only injures kidney tissue, but it also shifts the tissue away from repair, while L-carnitine pretreatment helps partially in maintaining greater kidney resilience under metal stress.

## 1. Introduction

Aluminium (Al), the most abundant metal in the Earth’s crust, has shifted from a naturally occurring geological element to an environmental contaminant of increasing concern. Although it is naturally present in soil and water, anthropogenic activities—including mining, acid rain-driven mobilisation, and the widespread use of aluminium in packaging, water treatment, and pharmaceuticals—have increased its bioavailable levels in the environment [1,2,3]. Under normal physiological conditions, trace amounts of absorbed Al are efficiently excreted by the kidneys, thereby maintaining systemic homeostasis. This balance, however, is fragile. As environmental burden rises or exposure intensifies, renal clearance may become insufficient, turning a low-level background exposure into a significant toxicological challenge [4]. This risk is particularly relevant under conditions of heightened exposure and in susceptible individuals with impaired renal clearance [5].

In renal tissue, oxidative stress and mitochondrial dysfunction are likely to be central drivers of Al-induced damage [6]. In this context, Al ions promote the generation of reactive oxygen species (ROS), leading to lipid peroxidation and DNA damage, which in turn activate the intrinsic apoptotic pathway [7]. This cascade culminates in the cleavage and activation of caspase-3, a key executioner of programmed cell death [8]. Although high-burden Al exposure is well established to induce apoptotic renal injury [9,10], the histopathological and functional outcome depends not only on the extent of apoptotic activation and structural damage, but also on the capacity of surviving tubular epithelium to initiate a proliferative response. Consequently, concurrent evaluation of injury-associated and proliferation-associated readouts can better define the integrated cortical response to severe Al stress than assessment of either endpoint alone.

The proliferative dimension of this response can be assessed using cell cycle markers such as Ki-67, a nuclear antigen expressed during active phases of the cell cycle (G_1_, S, G_2_, and M) and widely used as an indicator of cellular proliferation [11]. In the present study, the term ‘renal cortical injury–repair balance’ is used to describe the integrated relationship among three key readouts: structural injury (assessed by histopathology and quantitative histochemistry), apoptotic activation (quantified by cleaved caspase-3 immunoreactivity), and proliferative engagement (measured by Ki-67 labelling index).

A focused comparison of prior aluminium chloride (AlCl_3_) nephrotoxicity studies reveals a methodological gap in the integrated assessment of renal cell-dynamic readouts. Several investigations have documented AlCl_3_-induced apoptosis using cleaved caspase-3 immunoreactivity, including Sedik et al. [9], Yavuz et al. [10], Hassan et al. [12], and Rai et al. [13], but did not include Ki-67-labelled proliferative assessment. Conversely, studies that quantified cellular proliferation using Ki-67 labelling, including El-Kenawy et al. [14] and Domiaty [15], either did not assess apoptosis or did not evaluate cleaved caspase-3 concurrently. Therefore, to our knowledge, no previous AlCl_3_ nephrotoxicity study has concurrently evaluated cleaved caspase-3 immunoreactivity and Ki-67 labelling within the same renal cortical compartment under severe Al stress. Addressing this gap can improve understanding of how severe Al toxicity disrupts the relationship between epithelial injury and proliferative engagement.

L-carnitine (LC), an endogenous metabolite essential for mitochondrial fatty-acid transport and cellular energy metabolism [16,17,18], represents a plausible protective candidate. In experimental renal injury, LC has been reported to reduce oxidative stress, inflammatory signalling, and apoptotic activation [17]. To date, only one primary rat study has directly combined AlCl_3_ and LC while reporting kidney-specific outcomes [19]. That study showed improvement in serum urea and creatinine together with qualitative histological amelioration, but it did not determine whether LC meaningfully shifts the renal injury–repair balance by linking apoptotic activation to Ki-67-labelled proliferative engagement.

To establish a reproducible high-burden renal stress model, we adopted an oral AlCl_3_ regimen of 100 mg/kg body weight based on Thirunavukkarasu et al. [20], who demonstrated that this dose induces renal aluminium accumulation, functional impairment, oxidative stress, HSP70 activation, and histological kidney injury in male Wistar rats. This regimen was selected as a controlled experimental stressor for investigating severe Al-induced renal toxicity.

Accordingly, the present study aimed, for the first time, to examine the consequences of high-dose oral AlCl_3_ exposure on the renal injury–repair balance and to evaluate the protective potential of LC pretreatment in male Wistar rats. To address this aim, we used an integrative phenotyping framework combining functional, structural, histochemical, and cell-dynamic assessments in the same renal cortex, with particular emphasis on paired quantification of cleaved caspase-3 and Ki-67 as complementary markers of apoptotic activation and proliferative engagement.

## 2. Materials and Methods

### 2.1. Ethical Approval and Animals

All procedures were approved by the Ain Shams University Research Ethics Committee (ASU-SCI/ZOOL/2022/10/1). Experiments were conducted in accordance with the Guide for the Care and Use of Laboratory Animals (8th ed.) and the AVMA Guidelines for the Euthanasia of Animals (2020), as well as ARRIVE 2.0. Twenty male Wistar albino rats (*Rattus norvegicus*), two months old and weighing 98–144 g at baseline, were obtained from the Medical Research Centre, Faculty of Medicine, Ain Shams University, Cairo, Egypt.

Animals were acclimated for seven days before experimentation. Rats were randomly assigned and group-housed in plastic cages with environmental enrichment (nesting material). Each experimental group was distributed across two cages (three rats in one cage and two rats in the other) to minimise potential cage effects. Animals were maintained under a 12 h light:12 h dark cycle at 25 °C and 55–65% relative humidity, with ad libitum access to standard laboratory chow and tap water.

Animal care followed standard operating procedures. Daily welfare assessments during the dosing period included posture, activity, grooming, food and water intake, and clinical signs. Humane endpoints were predefined as ≥20% body-weight loss from baseline, body condition score ≤ 2/5, failure to eat or drink for >24 h, severe distress, or moribund state. No animals reached these predefined humane endpoints during the study.

Twenty-four hours after the final treatment administration on day 31, rats were anaesthetised with isoflurane to a surgical plane, confirmed by loss of pedal reflex. Following anaesthesia, euthanasia was performed by cervical dislocation by trained personnel. Death was confirmed by cessation of respiration and heartbeat. As this was a terminal non-recovery protocol, post-procedural analgesia was not required, and no animals were re-used in other experiments.

### 2.2. Chemicals and Experimental Design

LC capsules containing 350 mg LC powder per capsule were acquired from Pharmaceuticals & Medicinal Plants Arab Company, Enshas El Rami, Sharkeya, Egypt, and were manufactured by El-Nasr Pharmaceutical Chemicals Co. (Cairo, Egypt). Powdered aluminium chloride (AlCl_3_) was purchased from Alpha Chemika (Mumbai, Maharashtra, India). All reagents were of analytical grade. Both LC and AlCl_3_ were freshly prepared each day in sterile 0.9% saline immediately before oral gavage. For each rat, the required amount of LC or AlCl_3_ was calculated individually according to its current body weight to achieve the intended daily doses of 200 mg/kg for LC and 100 mg/kg for AlCl_3_. The calculated amount for each rat was dissolved in a final administration volume of 1 mL saline, and each rat received its corresponding 1 mL preparation by oral gavage. Therefore, the final working concentration varied according to individual body weight. Based on the baseline body-weight range of 98–144 g, the initial working concentrations were approximately 19.6–28.8 mg/mL for LC and 9.8–14.4 mg/mL for AlCl_3_. These working concentrations were below the reported aqueous solubility limits of both compounds. The preparations were mixed by gentle manual inversion until visually homogeneous and administered immediately. No vortexing, sonication, heating, or pH adjustment was required, and no visible precipitation or undissolved particles were observed before administration.

Following acclimation, male Wistar rats were randomly assigned to one of four experimental groups (*n* = 5 per group) using a computer-generated random number sequence. The treatment regimens, administered orally by gavage once daily for 30 consecutive days, were as follows:

1. Control, vehicle only (0.9% saline);
2. LC, 200 mg·kg^−1^ body weight;
3. AlCl_3_, 100 mg·kg^−1^ body weight;
4. Pretreatment group (LC+AlCl_3_), LC 200 mg·kg^−1^ followed 60 min later by AlCl_3_ 100 mg·kg^−1^.

Daily oral gavage of AlCl_3_ (100 mg·kg^−1^·day^−1^) for 30 consecutive days was used as a subacute renal exposure model. The high AlCl_3_ dose used has been reported to induce renal aluminium accumulation, functional renal impairment, oxidative stress, HSP70 activation, and histological kidney injury in male Wistar rats [20]. The LC dose (200 mg·kg^−1^·day^−1^) was chosen based on Zheng et al. [21], who observed robust renoprotective effects over four weeks without obvious toxicity; in the present study, we adopted the same daily dose over 30 days to align with the AlCl_3_ exposure window. The 60 min interval between LC and AlCl_3_ administration was prespecified to allow LC gastrointestinal absorption and systemic distribution before aluminium challenge. Animals were observed twice daily for general health and clinical signs throughout the dosing period, and no dose reductions were required.

### 2.3. Sample Collection and Biochemical Analysis

Twenty-four hours after the final dose, rats were weighed, anaesthetised, euthanised, and sampled. Blood was collected into plain tubes, allowed to clot, and centrifuged at 3000 rpm for 20 min, and serum was stored at −20 °C. Body-weight gain was calculated as terminal minus initial body weight.

Serum urea (mg/dL) and creatinine (mg/dL), serving as indices of renal function, were quantified using enzymatic colourimetric assays with commercial kits (Biodiagnostics, Dokki, Egypt) according to the manufacturer’s instructions. Measurements were performed on a Cobas automated clinical chemistry analyser (Roche Diagnostics, Mannheim, Germany), with two-level internal quality controls included in each run to ensure assay performance.

### 2.4. Histology and Lesion Scoring

For histological examination, one kidney from each rat, alternating left and right between animals to avoid systematic bias, was fixed in 10% neutral-buffered formalin for 24 h. Following fixation, tissues were processed by standard paraffin-embedding procedures, including dehydration in an ascending ethanol series, clearing in xylene, and embedding in paraffin wax. Paraffin blocks were sectioned at 5 μm, and sections were deparaffinised in xylene and rehydrated through a descending ethanol series to distilled water. Routine histology was performed using Harris’s haematoxylin and 1% aqueous eosin. Histological evaluation was performed by a single observer blinded to treatment allocation. Cortical injury was graded using a semi-quantitative 0–3 scale (0 = none, 1 = mild, 2 = moderate, and 3 = severe) for the following predefined lesions: (1) glomerular shrinkage, (2) dilatation of Bowman’s space, (3) vascular congestion, (4) vacuolar degeneration of tubular epithelial cells, (5) necrosis of tubular epithelial cells, (6) nuclear pyknosis of tubular epithelial cells, and (7) interstitial inflammatory cell infiltration. For each animal, at least 10 non-overlapping cortical fields at 400× magnification were examined, and a single 0–3 score was assigned to each lesion category based on the overall extent of change across fields. A composite histopathology score (range 0–21) was then calculated for each animal as the sum of the seven lesion scores and used as a secondary summary measure of total H&E-defined cortical injury burden. Individual lesion-domain scores were retained and reported separately for lesion-specific interpretation.

### 2.5. Histochemical Staining and Image Analysis

Periodic acid–Schiff, mercuric bromophenol blue, and Masson’s trichrome staining were used to assess cortical tubular microarchitecture, total protein distribution, and collagen deposition, respectively.

Histochemical staining for all experimental groups was performed in parallel batches to minimise inter-run variability. All sections were deparaffinised, rehydrated through a graded ethanol series, and processed using identical reagent timing and wash conditions within each stain. After staining, slides were imaged using a digital camera mounted on a Leica DM LS2 microscope (Leica Microsystems, Wetzlar, Germany) at the Mycology and Biotechnology Regional Centre (Al-Azhar University). Illumination, exposure, white balance, and magnification were held constant across groups using fixed non-automatic acquisition settings. The microscope lamp was allowed to stabilise before imaging, and a stage micrometre was used to verify spatial calibration at each magnification. Images were captured in RGB at identical resolution and saved as uncompressed TIFF files.

Quantitative image analysis was performed in Fiji (ImageJ) (version 1.54p) by a single observer blinded to treatment allocation, using a standardised workflow applied uniformly across all images. Spatial calibration (µm per pixel) was set in Fiji from the stage micrometre and applied to all measurements; the calibration value at 400× was approximately 0.53 µm per pixel. For each animal, five non-overlapping renal cortical fields were acquired at 400× from a single cortex-containing section per animal (5 µm thickness) using systematic random sampling, with a random start within cortex followed by a fixed step to ensure non-overlap. Fields were excluded a priori only for objective technical artefacts, including folds, tears, edge effects, or incomplete staining. Field-level measurements were averaged to yield one per-animal value per endpoint for inferential statistics. All analysis parameters, including segmentation rules and thresholds, were prespecified, locked prior to unblinding, and applied identically across groups. For quantification, RGB data were handled using a consistent 8-bit analysis pipeline, while original RGB TIFFs were retained as immutable source files.

#### 2.5.1. PAS Staining and Quantification Details

PAS staining was used to visualise PAS-positive carbohydrate-rich structures in the renal cortex, including tubular brush borders, apical glycocalyx, and basement membrane-rich components. Rehydrated sections were oxidised in periodic acid, rinsed, incubated with Schiff reagent, washed thoroughly in running tap water to develop the magenta reaction product, lightly counterstained with haematoxylin, dehydrated, cleared, and mounted.

Quantification was restricted to cortical parenchyma with exclusion of the capsule and large vessels. Glomerular profiles were excluded a priori from all PAS quantification to emphasise tubular microarchitecture.

PAS signal was quantified from RGB images using an 8-bit magenta-excess index computed pixel-wise as:

Magenta-excess = ((R + B)/2) − G,

where R, G, and B denote red, green, and blue channel intensities (0–255; arbitrary units). Magenta-excess was used as an operational colour-separation metric reflecting PAS-positive magenta staining intensity within carbohydrate-rich renal cortical structures. Analyses were restricted to tissue-only masks to exclude background and tubular lumina. Tissue masks were generated using HSV-based intensity segmentation to exclude low-saturation, high-value background, followed by standardised morphological clean-up including hole filling and removal of small debris. Lumina were defined as non-tissue voids fully enclosed by tissue; border-touching lumina were excluded. A fixed minimum lumen area filter of ≥100 pixels, approximately 28 µm^2^ at 0.53 µm/pixel, was applied to remove small voids and noise; this criterion was defined once from five representative fields and then applied unchanged to all images.

The following endpoints were extracted:

(a) Strong PAS area (%), defined as the percentage of tissue pixels with magenta-excess ≥60;
(b) Mean PAS magenta-excess, calculated across all tissue pixels;
(c) Rim magenta-excess mean, measured along a luminal rim defined as a fixed-width annulus extending from the detected lumen boundary into adjacent epithelium to index brush border-weighted PAS signal;
(d) Rim integrity (%), defined as the percentage of eligible lumina whose rim mean magenta-excess was ≥60.

Rim integrity was interpreted as an operational index of the continuity and preservation of the PAS-positive apical tubular rim; reduced rim integrity, therefore, indicated disruption or loss of the normally continuous PAS-positive tubular border, which is consistent with tubular microarchitectural injury.

Rim thickness was defined in physical units as approximately 2.6 µm and implemented as a 5-pixel annulus at the fixed 400× acquisition resolution based on the stage-micrometre calibration. The magenta-excess threshold (60) and rim definition were prespecified and applied identically across all groups. Lumina that could not be reliably segmented, such as collapsed profiles or truncated lumina, were excluded using the same objective rules for all images.

#### 2.5.2. Mercuric Bromophenol Blue Staining and Quantification Details

Mercuric bromophenol blue staining was used to visualise total protein distribution within the renal cortex. Following deparaffinisation and rehydration, sections were incubated in bromophenol blue working solution under standardised timing, rinsed, dehydrated, cleared, and coverslipped. Protein signal was quantified from bromophenol blue-stained images as mean optical density (OD) within cortical tubular tissue regions after exclusion of background and lumina using a tissue mask. Mean 8-bit intensity (I) was converted to OD as:

OD = log_10_(255/(I + 1)),

where the +1 term prevents division by zero. Regions of interest were restricted to cortex and preferentially sampled tubular parenchyma; where feasible, glomeruli and large vessels were excluded to emphasise tubulointerstitial protein signal.

#### 2.5.3. Masson’s Trichrome Staining and Collagen Quantification Details

Masson’s trichrome staining was used to assess collagen deposition and remodelling in the renal cortex. After rehydration, sections underwent sequential staining to differentiate nuclei, cytoplasm, and collagen fibres, with collagen visualised as blue or blue-green depending on formulation, followed by dehydration, clearing, and mounting. Collagen was quantified as area fraction (%) within standardised cortical regions of interest. Collagen-positive signal was isolated in Fiji using colour deconvolution by the Ruifrok–Johnston method with a single fixed stain matrix determined once from representative trichrome images to optimise separation of the collagen-associated, aniline-blue-weighted component and then applied unchanged to all images. The collagen component was thresholded using a single predefined rule applied uniformly across groups; threshold parameters were fixed before group comparisons and not adjusted post hoc. Collagen area fraction was calculated as collagen-positive pixels divided by total tissue pixels within the ROI, with background and lumina excluded by the tissue mask.

### 2.6. Immunohistochemistry for Cleaved Caspase-3 and Ki-67

Paraffin-embedded kidney sections (5 μm) were deparaffinised, rehydrated, and processed for immunohistochemical detection of cleaved caspase-3 and Ki-67 using an avidin–biotin–peroxidase complex (ABC) method, as previously described [22]. Endogenous peroxidase activity was blocked with 3% hydrogen peroxide in methanol for 10 min. Non-specific binding was minimised by incubating sections in 5% normal goat serum in phosphate-buffered saline for 30 min at room temperature. Primary antibodies were applied overnight at 4 °C in a humidified chamber. Cleaved caspase-3 was detected using a mouse monoclonal antibody (clone 2F7B8, Proteintech #68773-1-IG; 1:2000 dilution, citrate retrieval) on paraffin-embedded rat kidney sections. Ki-67 was detected using a mouse monoclonal antibody (clone 4A8, Boster #M00254-7; 1:500 dilution). After washing, sections were incubated with the appropriate biotinylated secondary antibody, followed by streptavidin–horseradish peroxidase. Immunoreactivity was visualised with 3,3′-diaminobenzidine as chromogen, yielding a brown reaction product, and nuclei were counterstained with Mayer’s haematoxylin. Negative controls were processed in parallel with omission of the primary antibody.

All quantitative immunohistochemistry analyses were restricted to the renal cortex. For each animal, 10 non-overlapping high-power fields at 400× magnification were selected using systematic uniform random sampling across a predefined cortical grid to ensure even coverage. Fields were excluded a priori if affected by section edges or technical artefacts, including folds, tears, uneven counterstaining, or non-specific background. Evaluations were performed by an observer blinded to experimental group allocation. Where feasible, glomeruli and large vessels were excluded to emphasise tubular cortical parenchyma. Cleaved caspase-3 immunoreactivity was assessed in cortical tubular epithelial cells, where brown DAB precipitate within the cytoplasm was considered positive staining, with occasional perinuclear or nuclear accentuation in apoptotic cells. In each 400× cortical field, the proportion of immunopositive tubular epithelial cells was graded as 0 (0%), 1 (1–25%), 2 (26–50%), 3 (51–75%), or 4 (76–100%). Staining intensity was scored as 0 (negative), 1 (mild), 2 (moderate), or 3 (strong). For each field, a semi-quantitative immunoreactivity index was calculated as the product of the proportion grade and intensity score, yielding a value from 0 to 12. The cleaved caspase-3 immunoreactivity index for each animal was expressed as the mean index across the 10 fields analysed. Animals with no detectable cleaved caspase-3 immunoreactivity across the evaluated cortical fields were assigned an index of 0.

Ki-67 expression was quantified as a labelling index, defined as the percentage of Ki-67-positive tubular epithelial nuclei, using a manual high-power counting approach adapted from El-Kenawy et al. [14] for renal cortex analysis. Sections were first screened at low magnification to identify well-preserved cortical regions with clear immunoreactivity, then examined at 400×. For each animal, 10 non-overlapping cortical fields were selected. In each field, 100 tubular epithelial nuclei were counted, and nuclei exhibiting brown DAB staining were recorded as Ki-67-positive. The Ki-67 labelling index for each field was calculated as the percentage of Ki-67-positive nuclei among the 100 nuclei counted. The value for each animal was expressed as the mean labelling index across the 10 fields. Animals with no detectable Ki-67 immunostaining across the evaluated cortical fields were assigned a labelling index of 0%.

### 2.7. Statistical Analysis

All analyses were performed in R (version 4.5.2), with the individual animal as the experimental unit. For image-derived endpoints, field-level measurements were averaged to one per-animal value before inference. Assumptions for parametric analysis were evaluated using graphical diagnostics together with the Shapiro–Wilk and Brown–Forsythe tests. Parametric data were analysed by one-way ANOVA followed by Tukey-adjusted post hoc comparisons, whereas nonparametric or ordinal data were analysed using the Kruskal–Wallis test followed by Dunn’s post hoc test with Bonferroni adjustment. Parametric data are presented as mean ± SD and nonparametric data as median (interquartile range). All tests were two-tailed, statistical significance was set at *p* < 0.05, and omnibus effect sizes were reported as ω^2^ for ANOVA and ε^2^ for Kruskal–Wallis tests.

## 3. Results

### 3.1. Body-Weight Gain

Body-weight gain differed among groups (one-way ANOVA, *F*_3,16_ = 47.43, *p* = 3.47 × 10^−8^, ω^2^ = 0.874), with mean values of 99.8 ± 8.6 g in control, 100.4 ± 12.6 g in LC, 24.0 ± 8.3 g in AlCl_3_, and 70.0 ± 15.6 g in LC+AlCl_3_ (Figure 1A).

### 3.2. Serum Biochemical Markers of Renal Function

Serum urea differed among groups (Figure 1B). One-way ANOVA showed a treatment effect (*F*_3,16_ = 58.89, *p* = 7.26 × 10^−9^, and ω^2^ = 0.897). The AlCl_3_ group showed the highest urea concentration (48.60 ± 5.81 mg/dL), exceeding control (26.40 ± 3.21 mg/dL), LC (23.18 ± 2.20 mg/dL), and LC+AlCl_3_ (21.40 ± 2.30 mg/dL) (Tukey-adjusted *p* ≤ 2.96 × 10^−7^ for AlCl_3_ versus each comparator). No significant differences were detected among control, LC, and LC+AlCl_3_ (all *p* ≥ 0.181). Pairwise standardised mean differences were large for AlCl_3_ versus LC+AlCl_3_ (Hedges’ *g* = 5.56, bootstrap 95% CI 4.09–15.65) and AlCl_3_ versus control (*g* = 4.27, bootstrap 95% CI 3.15–10.58).

Creatinine also differed among groups (Figure 1C). Because residual normality was not satisfied on the raw scale, ANOVA was performed on log-transformed creatinine. Group differences were significant (*F*_3,16_ = 17.64, *p* = 2.50 × 10^−5^, ω^2^ = 0.714). Post hoc testing showed higher creatinine in AlCl_3_-exposed rats (0.956 ± 0.147 mg/dL) than in control (0.606 ± 0.063 mg/dL), LC (0.594 ± 0.063 mg/dL), and LC+AlCl_3_ (0.580 ± 0.084 mg/dL) groups (Tukey-adjusted *p* ≤ 1.98 × 10^−4^), whereas control, LC, and LC+AlCl_3_ did not differ (*p* ≥ 0.932). Effect sizes on the inferential scale were large for AlCl_3_ versus LC+AlCl_3_ (*g* = 3.16, bootstrap 95% CI 2.53–6.93) and AlCl_3_ versus control (*g* = 3.27, bootstrap 95% CI 2.76–8.10).

### 3.3. Histological Assessment

Light microscopy of H&E-stained renal cortical sections showed group-dependent differences in glomerulotubular architecture. In the control group, glomeruli were normocellular with intact capillary tufts and a narrow Bowman’s space, and the surrounding proximal and distal convoluted tubules showed preserved cuboidal epithelium with patent lumina and no overt degenerative changes or interstitial inflammatory infiltrates (Figure 2A). Kidneys from LC-treated rats were morphologically comparable to controls (Figure 2B). In contrast, the AlCl_3_ group showed glomerular tuft contraction with widened urinary space, vascular congestion, tubular epithelial vacuolar degeneration, pyknotic nuclei, necrotic tubular profiles, and focal interstitial inflammatory cell accumulation (Figure 2C). In the LC+AlCl_3_ group, these lesions were less conspicuous than in the AlCl_3_ group, although mild residual tubular epithelial pallor and vacuolation remained visible in some profiles (Figure 2D). Photomicrographs were representative of the predominant histological pattern observed within each group.

Semi-quantitative H&E injury scoring showed significant group differences across all seven cortical lesion domains. AlCl_3_ exposure was associated with higher injury grades for glomerular shrinkage, urinary-space dilatation, vascular congestion, inflammatory cell infiltration, tubular vacuolar degeneration, epithelial necrosis, and nuclear pyknosis (Figure 3A), whereas control and LC groups remained at or near baseline (median 0). Omnibus group effects were significant for all lesion domains (H(3) = 11.62–17.26, *p* = 0.0088–6.26 × 10^−4^), with large effect sizes (ε^2^ = 0.539–0.891; bootstrap 95% CIs in Table 1). The LC pretreatment group showed lower scores than AlCl_3_ for vascular congestion, inflammatory infiltration, and tubular vacuolar degeneration (Figure 3A). However, for glomerular shrinkage, urinary-space dilatation, and tubular necrosis, the LC pretreatment group showed intermediate median scores that did not differ from AlCl_3_ after Dunn–Bonferroni correction.

To integrate the ordinal H&E lesion profile into a single endpoint, the seven cortical lesion scores were summed per animal to yield a composite cortical injury burden score (range 0–21) (Figure 3B). Composite scores differed among groups (Kruskal–Wallis *H*(3) = 17.61, *p* = 5.28 × 10^−4^), with a large effect size (ε^2^ = 0.913, bootstrap 95% CI 0.910–0.977). Control animals exhibited a composite score of 0 (0–0), the LC group 1 (0–1), the AlCl_3_ group 15 (15–15), and the LC+AlCl_3_ group 3 (3–4).

### 3.4. Histochemical Assessment

Histochemical staining was performed to assess carbohydrate-rich structures by PAS, tissue protein distribution by mercuric bromophenol blue, and collagen deposition by Masson’s trichrome.

#### 3.4.1. PAS Histochemistry: Tubular Microarchitecture and Apical PAS Rim Integrity

PAS staining revealed treatment-related differences in cortical tubular PAS-positive microarchitecture, including the brush border-weighted apical PAS rim. In the control group (Figure 4A), cortical tubules showed dense PAS reactivity with a crisp, continuous apical rim along proximal convoluted tubules, together with expected PAS positivity in basement membrane-rich structures and glomerular tufts. The LC group (Figure 4B) was broadly comparable to controls. In contrast, the AlCl_3_ group (Figure 4C) showed generalised PAS pallor with weakened PAS-positive tubular profiles and frequent rim fragmentation or loss. The LC+AlCl_3_ group (Figure 4D) showed partial preservation of tubular PAS signal and detectable apical rim segments, although rim continuity remained reduced relative to controls.

Quantitative Fiji-derived indices corroborated the microscopic pattern. Strong PAS area (%) differed among groups (one-way ANOVA on logit-transformed percentages, *F*_3,16_ = 54.64, *p* = 1.25 × 10^−8^, and ω^2^ = 0.889). Strong PAS area was 97.92 ± 1.10% in control, 91.15 ± 4.05% in LC, 52.37 ± 14.68% in AlCl_3_, and 89.25 ± 2.67% in LC+AlCl_3_ (Figure 5A). AlCl_3_ differed from control, LC, and LC+AlCl_3_ (all Tukey-adjusted *p* < 0.0001). Control exceeded LC (*p* = 6.0 × 10^−4^) and LC+AlCl_3_ (*p* = 1.0 × 10^−4^), whereas LC and LC+AlCl_3_ did not differ (*p* = 0.799).

Mean magenta-excess also showed a group effect (one-way ANOVA, *F*_3,16_ = 62.47, *p* = 4.72 × 10^−9^, and ω^2^ = 0.902). Mean magenta-excess was 127.36 ± 4.98 a.u. in control, 105.69 ± 11.71 a.u. in LC, 54.41 ± 10.32 a.u. in AlCl_3_, and 95.18 ± 5.57 a.u. in LC+AlCl_3_ (Figure 5B). AlCl_3_ differed from control, LC, and LC+AlCl_3_ (all Tukey-adjusted *p* < 0.0001). Control exceeded LC (*p* = 0.0056) and LC+AlCl_3_ (*p* = 1.0 × 10^−4^), whereas LC and LC+AlCl_3_ did not differ (*p* = 0.259).

Rim magenta-excess mean showed the largest separation among groups (one-way ANOVA, *F*_3,16_ = 82.96, *p* = 5.76 × 10^−10^, and ω^2^ = 0.925). Rim signal was 81.60 ± 2.85 a.u. in control, 63.96 ± 6.27 a.u. in LC, 16.21 ± 10.47 a.u. in AlCl_3_, and 58.23 ± 5.31 a.u. in LC+AlCl_3_ (Figure 5C). AlCl_3_ differed from control, LC, and LC+AlCl_3_ (all Tukey-adjusted *p* < 0.0001). Control exceeded LC (*p* = 0.0042) and LC+AlCl_3_ (*p* = 3.0 × 10^−4^), whereas LC and LC+AlCl_3_ did not differ (*p* = 0.558). Rim integrity showed a zero-inflated distribution and was therefore analysed nonparametrically (Kruskal–Wallis, *H*(3) = 17.00, *p* = 7.06 × 10^−4^, and ε^2^ = 0.875).

Rim integrity collapsed to zero in the AlCl_3_ group, whereas control retained near-ceiling values and LC and LC+AlCl_3_ showed intermediate central tendency (Figure 5D). Post hoc Dunn–Bonferroni comparisons showed a significant difference only between control and AlCl_3_.

#### 3.4.2. Mercuric Bromophenol Blue Histochemistry: Total Protein Microarchitecture

Mercuric bromophenol blue staining revealed treatment-related differences in renal cortical protein microarchitecture (Figure 6). In the control group (Figure 6A), cortical tubules displayed strong, relatively uniform cytoplasmic protein staining and glomerular tufts were readily identifiable. The LC group (Figure 6B) remained broadly comparable to controls. In contrast, the AlCl_3_ group (Figure 6C) showed attenuation of bromophenol blue staining with generalised cortical pallor, patchy tubular signal, epithelial clearing, and luminal dilatation in multiple profiles. The LC+AlCl_3_ group (Figure 6D) showed stronger tubular protein staining and less patchiness than the AlCl_3_ group, although focal variability persisted in some cortical regions.

Quantitative optical density analysis corroborated the qualitative pattern (one-way ANOVA, *F*_3,16_ = 14.09, *p* = 9.40 × 10^−5^, and ω^2^ = 0.663). Mean protein OD was 0.353 ± 0.020 in control, 0.337 ± 0.016 in LC, 0.269 ± 0.039 in AlCl_3_, and 0.354 ± 0.012 in LC+AlCl_3_ (Figure 5E). Tukey-adjusted comparisons showed that AlCl_3_ differed from control (*p* = 0.0002), LC (*p* = 0.0020), and LC+AlCl_3_ (*p* = 0.0002), whereas control, LC, and LC+AlCl_3_ did not differ (all *p* ≥ 0.67).

#### 3.4.3. Masson’s Trichrome Histochemistry: Collagen Deposition and Interstitial Remodelling

Masson’s trichrome staining revealed treatment-related differences in renal cortical collagen distribution (Figure 7A–D). In the control group (Figure 7A), collagen signal was limited to thin, discontinuous strands within expected interstitial compartments and peritubular boundaries. The LC group (Figure 7B) remained broadly comparable to controls. In contrast, the AlCl_3_ group (Figure 7C) showed expansion of collagen-positive signal within the cortical interstitium and peritubular regions, with thicker interstitial deposits and more extensive collagen-positive areas. The LC+AlCl_3_ group (Figure 7D) showed less collagen-positive expansion than the AlCl_3_ group, although collagen signal remained higher than in non-exposed cortex.

Quantitative collagen area fraction supported this pattern (one-way ANOVA on logit-transformed percentages, *F*_3,16_ = 32.79, *p* = 4.58 × 10^−7^, ω^2^ = 0.827, and bootstrap 95% CI 0.692–0.938). Collagen area fraction was 6.92 ± 1.67% in control, 6.02 ± 1.50% in LC, 18.40 ± 3.02% in AlCl_3_, and 12.26 ± 1.70% in LC+AlCl_3_ (Figure 5F). AlCl_3_ exceeded control and LC (both Tukey-adjusted *p* < 0.0001). LC+AlCl_3_ was lower than AlCl_3_ (*p* = 0.0229). Control and LC did not differ (*p* = 0.9919), whereas LC+AlCl_3_ exceeded control (*p* = 0.0021) and LC (*p* = 0.0003).

### 3.5. Immunohistochemistry: Cleaved Caspase-3 and Ki-67 Labelling

Cleaved caspase-3 and Ki-67 immunohistochemistry revealed group-dependent differences in cortical tubular labelling patterns.

In the control group (Figure 8A), cleaved caspase-3 immunoreactivity was minimal and largely confined to scattered tubular epithelial profiles, whereas Ki-67-positive tubular nuclei were detectable (Figure 9A). The LC group showed a similar pattern, with low cleaved caspase-3 immunoreactivity (Figure 8B) and detectable Ki-67 labelling (Figure 9B). In the AlCl_3_ group, cleaved caspase-3 immunoreactivity was prominent and widely distributed across cortical tubules (Figure 8C), whereas Ki-67 labelling was markedly reduced, with few Ki-67-positive tubular nuclei visible (Figure 9C). In the LC+AlCl_3_ group, cleaved caspase-3 immunoreactivity was less extensive than in the AlCl_3_ group (Figure 8D), and Ki-67-positive nuclei were more frequent than in the AlCl_3_ group (Figure 9D), although labelling remained below control levels.

Quantitatively, the cleaved caspase-3 composite index (0–12) differed among groups (Kruskal–Wallis, *H*(3) = 17.13, *p* = 6.65 × 10^−4^, ε^2^ = 0.883, and bootstrap 95% CI 0.864–0.974). Cleaved caspase-3 indices were 1.0 (1.0–2.0) in control, 2.0 (2.0–2.0) in LC, 12.0 (12.0–12.0) in AlCl_3_, and 4.0 (4.0–6.0) in LC+AlCl_3_ (Figure 10A). Dunn–Bonferroni comparisons showed higher cleaved caspase-3 scores in AlCl_3_ than in control and LC. AlCl_3_ scores reached the maximum value of the composite scale across animals. The Ki-67 labelling index (%) also differed among groups (one-way ANOVA, *F*_3,16_ = 31.25, *p* = 6.36 × 10^−7^, ω^2^ = 0.819, and bootstrap 95% CI 0.781–0.928). Ki-67 labelling was 17.80 ± 3.35% in control, 18.80 ± 2.17% in LC, 6.00 ± 1.58% in AlCl_3_, and 10.60 ± 2.30% in LC+AlCl_3_ (Figure 10B). Tukey-adjusted post hoc comparisons showed that AlCl_3_ was lower than control, LC, and LC+AlCl_3_, and that LC+AlCl_3_ was lower than control and LC.

## 4. Discussion

AlCl_3_ exposure in the present model produced a coherent renal injury phenotype, spanning from systemic growth suppression to overt cortical dysfunction and severe structural disruption. Systemic Al exposure promotes chronic multiorgan dysfunction and broad metabolic disruption, including impaired energy metabolism [23]. Accordingly, the marked attenuation of body-weight gain observed in AlCl_3_-treated rats suggests that the toxic burden was not restricted to a localised renal lesion, but instead reflected a systemic disturbance in which physiological resources were increasingly diverted towards maintaining homeostasis rather than supporting normal somatic growth. At the organ level, this systemic stress was accompanied by a pronounced rise in serum urea and creatinine, consistent with impaired renal clearance and substantial loss of functional integrity [20]. Histologically, this functional decline was mirrored by widespread glomerulotubular disruption—characterised by vascular congestion, interstitial inflammatory infiltration, tubular vacuolar degeneration, epithelial necrosis, and nuclear pyknosis—with semi-quantitative scoring confirming a markedly elevated cortical injury burden in the AlCl_3_-treated group. Beyond conventional morphology, histochemical analysis revealed deeper alterations in cortical tubular microarchitecture and tissue composition. PAS staining demonstrated profound disruption of glycoconjugate-rich tubular domains, with the most prominent deficit localised to the apical brush border, a pattern consistent with severe compromise of proximal tubular epithelial integrity [24]. Complementing these findings, bromophenol blue staining revealed reduced cortical protein intensity and loss of microarchitectural uniformity, indicating a broader disturbance of tubular cytoplasmic organisation; this pattern of protein depletion aligns with histochemical profiles reported in severe nephrotoxic models [25]. In parallel, Masson’s trichrome staining demonstrated an expansion of the collagen-positive cortical area, suggesting that AlCl_3_ injury had shifted towards early interstitial remodelling. When considered together with the strong increase in cleaved caspase-3 immunoreactivity and the marked decline in Ki-67 labelling, these findings support a shift in the cortical environment towards cell loss, limited renewal, and reduced structural resilience.

Building on these findings, a plausible integrative framework for these observations is mitochondrial dysfunction within the highly energy-dependent proximal tubular epithelium. Proximal tubular cells rely heavily on CPT1α-linked mitochondrial fatty-acid oxidation to sustain mitochondrial respiration, ATP production, and epithelial phenotype [26]. Although mitochondrial bioenergetics were not directly quantified in the present study, the observed injury pattern—particularly the loss of apical brush border integrity—is highly compatible with a state of bioenergetic failure. In this context, disruption of ATP-dependent cytoskeletal regulation can destabilise the cortical actin network and contribute to disassembly of brush border microvilli, thereby compromising epithelial polarity and tubular function [27]. AlCl_3_ was found to disrupt mitochondrial homeostasis and bioenergetic function in other tissues [28,29], supporting the biological plausibility of a similar contribution in the kidney under severe AlCl_3_ stress.

Such bioenergetic failure could also amplify oxidative stress, as impaired mitochondrial efficiency increases electron leakage and excess ROS generation, establishing a self-reinforcing cycle of mitochondrial dysfunction and redox injury [30]. In experimental AlCl_3_ nephrotoxicity, this process has been associated with increased lipid peroxidation together with depletion of key antioxidant defences, including SOD, GST, and GSH [20,31].

The resulting oxidative stress may plausibly promote secondary inflammatory amplification, thereby exacerbating tissue injury. Prior studies have reported that excess ROS can activate the NLRP3 inflammasome via the JNK pathway [32]. In parallel, AlCl_3_-induced oxidative stress can also activate NF-κB, a central regulator of inflammatory gene expression, thereby promoting production of pro-inflammatory cytokines such as TNF-α, IL-1β, and IL-6 [33]. Inflammasome activation further promotes maturation of inflammatory cytokines, including IL-1β and IL-18 [34], which may facilitate immune-cell recruitment and amplify renal tissue injury. In this context, the prominent interstitial inflammatory infiltration and vascular congestion observed histologically in the present study are consistent with an oxidative stress-associated inflammatory component contributing to cortical damage.

The central mechanistic insight in the present AlCl_3_ model was the marked imbalance between apoptotic activation and proliferative engagement in cortical tubules. Immunohistochemically, this was reflected by strong, widespread cleaved caspase-3 labelling across cortical tubules together with a marked reduction in Ki-67 labelling to ~6%, indicating that tubular injury was accompanied not only by active programmed cell loss but also by a reduced proliferative engagement.

A possible literature-based explanation for the marked increase in cleaved caspase-3 labelling is activation of the intrinsic mitochondrial apoptotic pathway. In this framework, ROS-induced DNA damage can promote p53 activation [7,35], which in turn enhances pro-apoptotic Bcl-2 family signalling, particularly through Bax, leading to mitochondrial cytochrome c release and downstream activation of caspase-9 and caspase-3 [8,36].

In striking contrast, Ki-67, a nuclear marker of active cell cycle engagement, was markedly reduced. Under normal conditions, tubular injury can trigger compensatory epithelial proliferation to restore structural integrity [37,38]; here, however, that reparative response appeared markedly blunted. Notably, the renal proliferative response to AlCl_3_ is not uniform but varies with injury severity. Under milder exposure conditions (34 mg/kg b.w.), AlCl_3_ significantly increased renal Ki-67 labelling relative to controls (99.112 ± 7.821 vs. 9.512 ± 3.331) [14], a pattern that may reflect compensatory epithelial proliferative activation. In the present higher-dose subacute model (100 mg/kg b.w.), however, the simultaneous increase in cleaved caspase-3 and suppression of Ki-67 suggests that the injury burden exceeded reparative capacity, shifting the cortex from compensatory proliferation towards dominant cell loss and suppressed proliferative renewal. This suppression may plausibly reflect sustained toxic stress, which can promote checkpoint activation and prolonged cell cycle arrest in injured tubular epithelium [39], thereby limiting progression through active proliferative phases [40,41,42]. In severe injury states, such arrest may initially be protective; however, persistent failure to re-enter the cell cycle is increasingly linked to maladaptive repair and progression towards fibrotic remodelling [43]. Collectively, these findings indicate that AlCl_3_ in the present study not only triggered epithelial cell death, but also compromised the kidney’s proliferative response.

This sustained apoptotic–proliferative imbalance provides a plausible biological context for the profibrotic shift observed in the AlCl_3_ group. Persistent epithelial injury and cell death can promote TGF-β1-driven fibrogenic signalling, leading to fibroblast activation, extracellular matrix accumulation, and progressive architectural distortion [44,45]. In this context, the significant collagen expansion detected here by Masson’s trichrome staining is consistent with an early maladaptive shift towards interstitial remodelling.

Against this injury backdrop, LC pretreatment conferred substantial but incomplete renoprotection. Relative to AlCl_3_ alone, LC pretreatment improved body-weight gain, normalised serum urea and creatinine, reduced cortical lesions, partially preserved PAS-positive apical domains and bromophenol blue staining, attenuated cleaved caspase-3, partially restored Ki-67, and limited collagen expansion. Collectively, these changes indicate that LC shifted the renal cortical response towards a more repair-permissive and structurally resilient state, rather than fully preventing toxic injury.

A plausible literature-based explanation is that LC preserved mitochondrial function and thereby attenuated the downstream cascade of oxidative, inflammatory, apoptotic, and profibrotic injury. By facilitating mitochondrial fatty-acid transport and ATP generation, LC may support epithelial bioenergetics and reduce excess ROS formation [46,47]. In parallel, LC has been reported to reinforce antioxidant defences, suppress inflammatory signalling, rebalance the Bax–Bcl-2 axis, and reduce downstream caspase activation [21,48]. Together, these actions provide a plausible basis for the improved microarchitecture, reduced inflammatory infiltration, and lower apoptotic burden observed in the LC+AlCl_3_ group.

This protective milieu may also help explain the partial restoration of proliferative engagement. LC has been reported to activate PI3K and AKT signalling, which promotes cell survival and proliferative capacity while suppressing pro-apoptotic signalling [21]. This effect is mechanistically important because tubular repair depends on the survival, dedifferentiation, proliferation, and re-establishment of epithelial continuity by residual tubular cells [49]. Accordingly, the current intermediate Ki-67 index suggests that LC pretreatment improved the conditions required for reparative cell cycle re-entry without fully normalising proliferative capacity.

Consistent with this interpretation, the reduction in collagen accumulation in the LC+AlCl_3_ group supports partial attenuation of profibrotic remodelling. A plausible literature-based mechanism is that LC interferes with TGF-β- and Smad-associated profibrotic signalling, potentially through regulators such as PPAR-γ and Sirt1, both of which can antagonise TGF-β1 signalling and suppress myofibroblast activation [50]. However, collagen content remained elevated relative to control levels, indicating that LC did not fully abolish fibrotic progression under continued AlCl_3_ challenge.

Taken together, the findings of the present study support a phenotype of renal functional impairment, cortical structural injury, PAS-positive tubular microarchitectural disruption, reduced protein staining, collagen expansion, increased cleaved caspase-3 immunoreactivity, and reduced Ki-67-labelled proliferative engagement following subacute high-dose AlCl_3_ exposure. This observed phenotype aligns with literature-reported mechanisms involving mitochondrial bioenergetic stress, oxidative stress amplification, inflammatory signalling, apoptosis-related pathways, cell cycle dysregulation, and early profibrotic responses. In contrast, LC pretreatment partially mitigated this injury phenotype by preserving renal function markers, maintaining tubular microarchitecture, reducing collagen deposition, lowering cleaved caspase-3 immunoreactivity, and partially restoring proliferative engagement, thereby shifting the renal cortical injury–repair balance toward greater structural and cellular preservation under AlCl_3_-induced stress.

## 5. Conclusions

In male Wistar rats, high-dose AlCl_3_ exposure (100 mg·kg^−1^·day^−1^ for 30 days) profoundly disrupted the renal injury–repair balance, whereas LC pretreatment (200 mg·kg^−1^·day^−1^) afforded substantial, but incomplete, protection by attenuating functional and structural injury, reducing apoptotic signalling, and partially restoring proliferative engagement. To our knowledge, this is the first high-burden AlCl_3_ study to quantify paired cleaved caspase-3 and Ki-67 within the same renal cortical compartment and to show that LC pretreatment modulates this injury–repair balance. Notably, the data indicate that severe Al nephrotoxicity is driven not only by enhanced cell loss but also by impaired regenerative renewal, distinguishing this model from milder exposure settings in which compensatory proliferation persists. LC therefore emerges as a pretreatment modulator that partially preserves cortical resilience under toxicant stress.

Future studies using larger cohorts, both sexes, direct oxidative-stress and mitochondrial assays, dose-ranging designs, and time-course sampling would further refine the protective framework by defining sex-dependent responses, mechanistic pathways, temporal progression, minimum effective dose, dose dependency, and the potential ceiling of LC protection under AlCl_3_-induced renal stress.

## Figures and Tables

**Figure 1:**
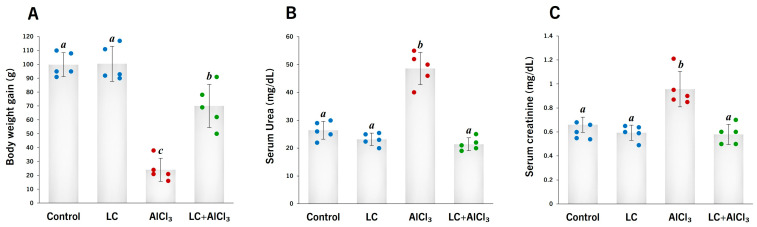
Growth and renal functional responses to AlCl_3_ stress and LC pretreatment. (**A**) Body-weight gain (g) over 30 days. (**B**) Serum urea (mg/dL). (**C**) Serum creatinine (mg/dL). Bars show mean ± SD with individual rats overlaid (*n* = 5 per group). Different letters indicate significant group differences (one-way ANOVA with Tukey post hoc, *p* < 0.05).

**Figure 2:**
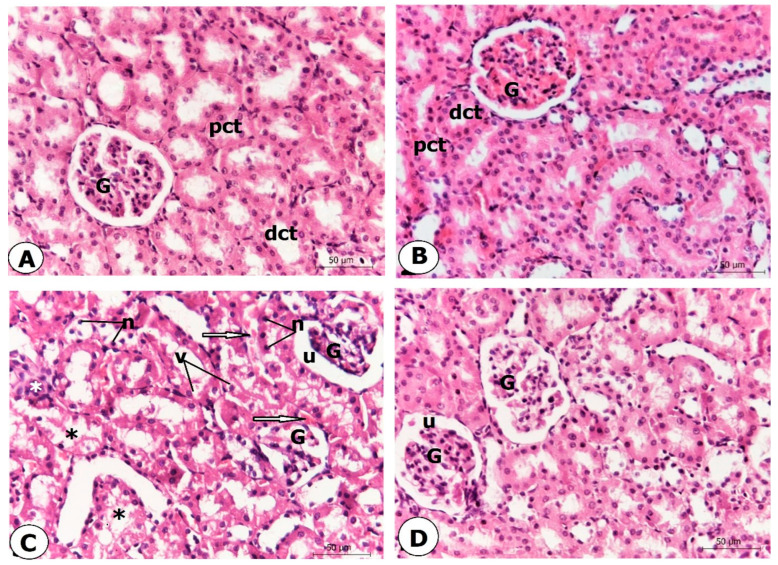
Representative photomicrographs of haematoxylin and eosin (H&E)-stained renal cortical sections from experimental groups (×400; scale bar = 50 μm). (**A**) Control cortex with preserved glomerulus (G), proximal convoluted tubules (pct), and distal convoluted tubules (dct). (**B**) LC cortex showing preserved cortical architecture. (**C**) AlCl_3_ cortex showing contracted glomerular tufts (G) with widened urinary space (u), vascular congestion (white arrows), tubular epithelial vacuolar degeneration (v), pyknotic nuclei (n), and necrotic tubules (black asterisks), with focal interstitial inflammatory cell accumulation (white asterisk). (**D**) LC+AlCl_3_ (pretreatment) cortex showing attenuation of AlCl_3_-associated lesions, with less prominent urinary-space widening (u) and comparatively preserved glomerular profiles (G) and tubular architecture.

**Figure 3:**
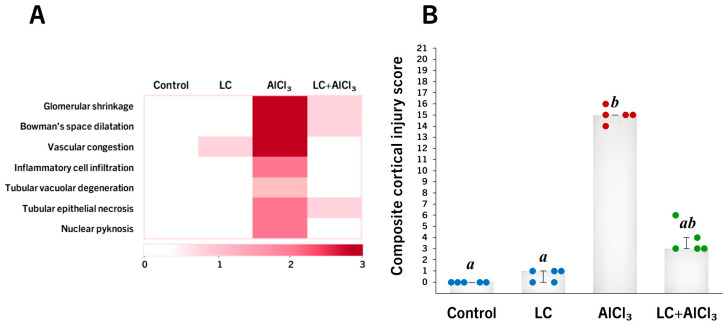
Cortical histopathology under AlCl_3_ and LC pretreatment. (**A**) Heatmap of median ordinal lesion scores (0–3; 0 = none, 1 = mild, 2 = moderate, and 3 = severe) across seven H&E-defined cortical injury domains (*n* = 5 per group). (**B**) Composite cortical injury score (0–21) calculated as the sum of the seven lesion scores per animal. Points represent individual rats; central tendency and dispersion are shown as median with interquartile range (Q1–Q3). Groups sharing the same letter do not differ significantly (Kruskal–Wallis with Dunn post hoc tests and Bonferroni correction, adjusted *p* ≥ 0.05).

**Figure 4:**
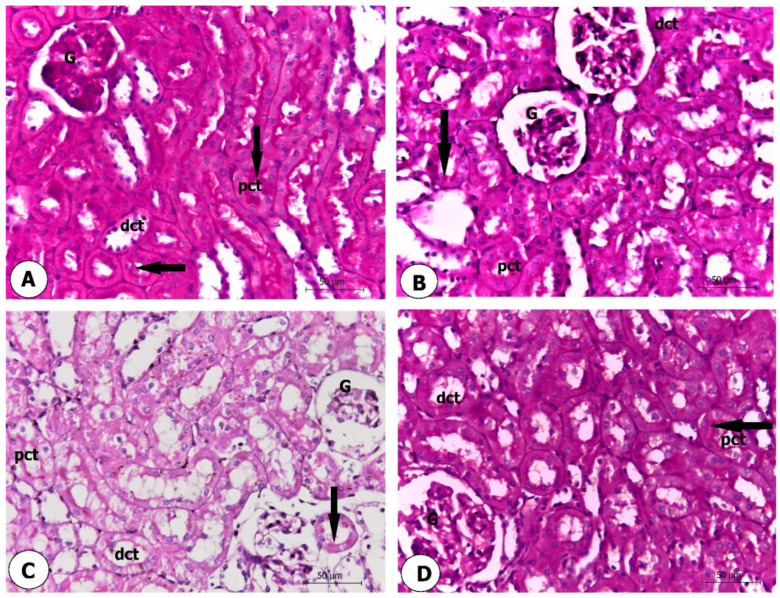
Periodic acid–Schiff (PAS) histochemistry of renal cortical tubular microarchitecture across experimental groups (×400; scale bar = 50 μm). (**A**) Control cortex showing strong PAS reactivity with a preserved apical PAS rim along proximal convoluted tubules (pct; arrows), glomerular tuft (G), and distal convoluted tubules (dct). (**B**) LC cortex showing preserved PAS staining. (**C**) AlCl_3_ cortex showing marked PAS pallor and loss of the apical rim signal (arrow). (**D**) LC+AlCl_3_ (pretreatment) cortex showing partial preservation of PAS-positive tubular profiles and detectable rim segments in pct (arrow).

**Figure 5:**
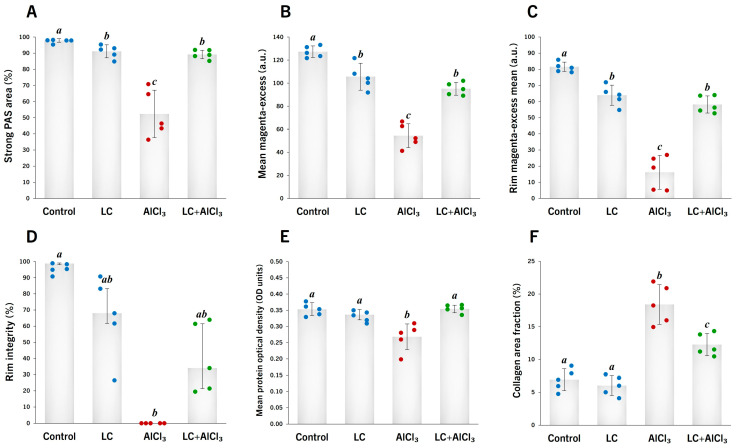
Quantitative histochemical indices of renal cortex under AlCl_3_ stress and LC pretreatment. (**A**) Strong PAS area (% tissue above threshold). (**B**) Mean magenta-excess (a.u.) across cortical tissue. (**C**) Rim magenta-excess mean (a.u.). (**D**) Rim integrity (% of eligible lumina meeting the rim threshold). (**E**) Bromophenol blue-derived mean protein optical density. (**F**) Masson’s trichrome-derived collagen area fraction. Bars show mean ± SD for panels (**A**–**C**,**E**,**F**), and median (IQR) for panel (**D**), with individual rats overlaid (*n* = 5 per group). Different letters indicate significant group differences ((**A**–**C**,**E**,**F**): one-way ANOVA with Tukey post hoc; (**D**): Kruskal–Wallis with Dunn–Bonferroni; *p* < 0.05).

**Figure 6:**
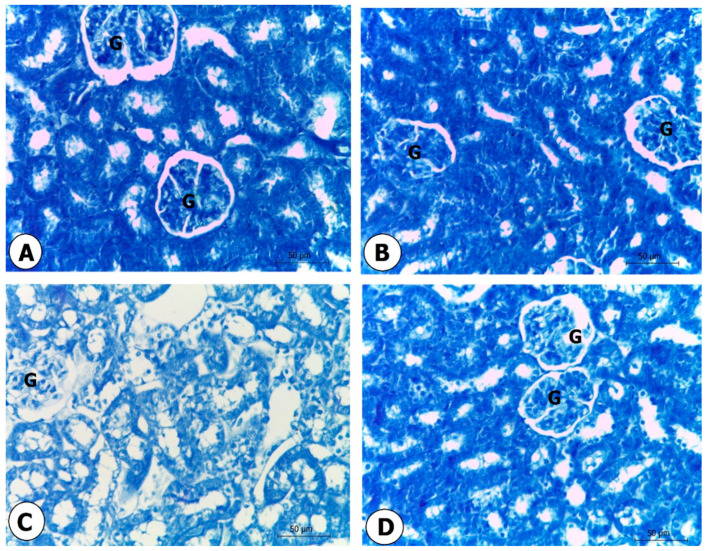
Mercuric bromophenol blue histochemistry of renal cortical protein microarchitecture across experimental groups (×400; scale bar = 50 μm)**.** (**A**) Control cortex showing strong, relatively uniform tubular cytoplasmic protein staining; glomeruli are indicated (G). (**B**) LC cortex showing preserved tubular protein signal comparable to control. (**C**) AlCl_3_ cortex showing reduced staining intensity, pallor, and patchy tubular protein signal, accompanied by prominent epithelial clearing and luminal dilatation in multiple profiles. (**D**) LC pretreatment cortex showing stronger tubular protein staining and less cortical patchiness than the AlCl_3_ panel, with residual focal heterogeneity.

**Figure 7:**
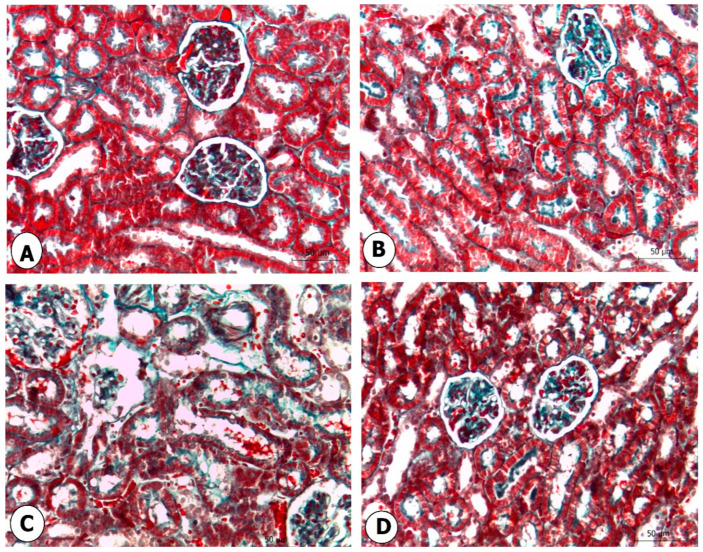
Masson’s trichrome histochemistry of renal cortical collagen distribution across experimental groups (×400; scale bar = 50 μm)**.** (**A**) Control cortex showing limited collagen signal (blue to blue-green) confined to expected interstitial compartments and peritubular boundaries with preserved cortical organisation. (**B**) LC cortex showing collagen distribution comparable to control. (**C**) AlCl_3_ cortex showing interstitial and peritubular collagen expansion. (**D**) LC pretreatment cortex showing a lower extent of collagen-positive expansion than the AlCl_3_ panel, although collagen staining remains more apparent than in non-exposed cortex.

**Figure 8:**
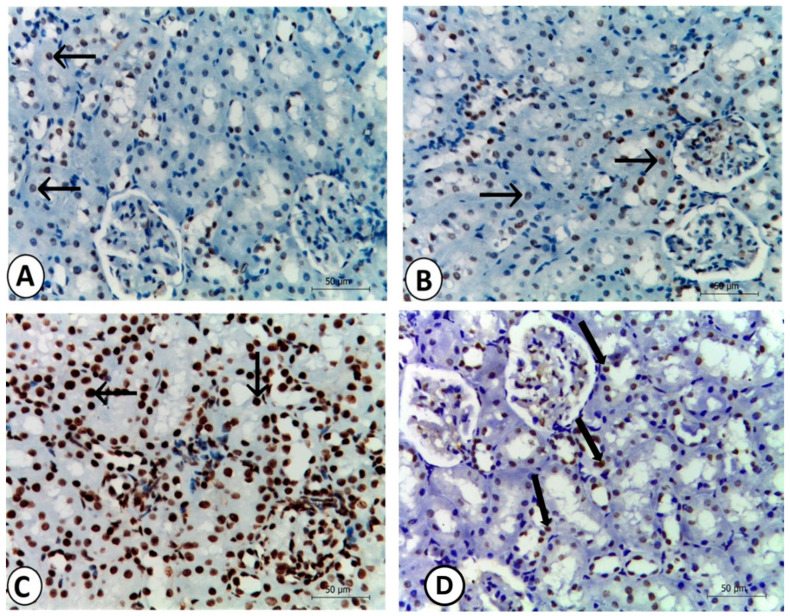
Cleaved caspase-3 immunohistochemistry in renal cortex across experimental groups (×400; scale bar = 50 μm). Representative cortical fields showing cleaved caspase-3 immunoreactivity in tubular epithelium (brown DAB) with haematoxylin counterstain. Arrows indicate examples of cleaved caspase-3-positive tubular epithelial staining. (**A**) Control. (**B**) LC. (**C**) AlCl_3_. (**D**) LC+AlCl_3_. AlCl_3_ induced strong, widespread tubular immunoreactivity, which was attenuated by LC pretreatment.

**Figure 9:**
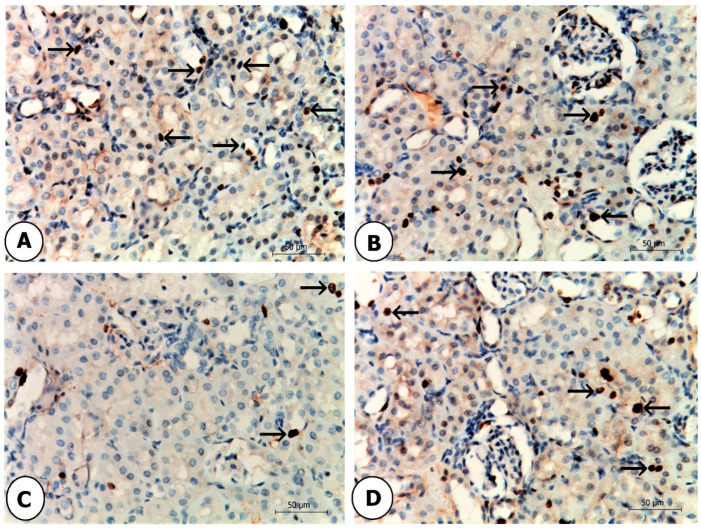
Ki-67 immunohistochemistry in renal cortex across experimental groups (×400; scale bar = 50 μm). Representative cortical fields showing Ki-67-positive tubular epithelial nuclei (brown DAB) with haematoxylin counterstain. Arrows indicate examples of Ki-67-positive nuclei. (**A**) Control. (**B**) LC. (**C**) AlCl_3_. (**D**) LC+AlCl_3_. Ki-67 labelling was markedly reduced by AlCl_3_ and partially restored by LC pretreatment.

**Figure 10:**
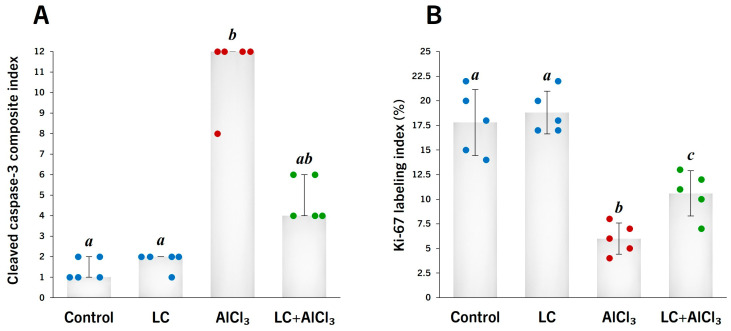
Cortical tubular cell-dynamic markers under AlCl_3_ stress and LC pretreatment. (**A**) Cleaved caspase-3 composite index (0–12), shown as median (IQR) with individual rats overlaid (*n* = 5 per group). (**B**) Ki-67 labelling index (%), shown as mean ± SD with individual rats overlaid (*n* = 5 per group). Different letters indicate significant group differences ((**A**): Kruskal–Wallis with Dunn–Bonferroni; (**B**): one-way ANOVA with Tukey; *p* < 0.05).

**Table 1:** Semi-quantitative H&E lesion scores in the renal cortex (ordinal 0–3; *n* = 5 per group).

Effect Size (ε^2^) (Bootstrap 95% CI)	*p*	Omnibus Test	LC+AlCl_3_	AlCl_3_	LC	Control	Lesion (Score 0–3)
0.886 (0.814–1.000)	6.50 × 10^−4^	Kruskal–Wallis H(3) = 17.18	1 (1–1) ᵃᵇ	3 (2–3) ᵇ	0 (0–0) ᵃ	0 (0–0) ᵃ	Glomerular shrinkage
0.891 (0.820–1.000)	6.26 × 10^−4^	Kruskal–Wallis H(3) = 17.26	1 (1–1) ᵃᵇ	3 (3–3) ᵇ	0 (0–0) ᵃ	0 (0–0) ᵃ	Urinary (Bowman’s) space dilatation
0.707 (0.695–0.898)	0.0025	Kruskal–Wallis H(3) = 14.31	0 (0–1) ᵃ	3 (3–3) ᵇ	1 (0–1) ᵃᵇ	0 (0–0) ᵃ	Vascular congestion
0.857 (0.814–1.000)	8.12 × 10^−4^	Kruskal–Wallis H(3) = 16.71	0 (0–0) ᵃ	2 (2–3) ᵇ	0 (0–0) ᵃ	0 (0–0) ᵃ	Inflammatory cell infiltration
0.792 (0.699–1.000)	0.0013	Kruskal–Wallis H(3) = 15.67	0 (0–0) ᵃ	1 (1–2) ᵇ	0 (0–0) ᵃ	0 (0–0) ᵃ	Tubular vacuolar degeneration
0.735 (0.613–0.936)	0.0020	Kruskal–Wallis H(3) = 14.76	1 (1–2) ᵃᵇ	2 (1–2) ᵇ	0 (0–0) ᵃ	0 (0–0) ᵃ	Tubular epithelial necrosis
0.539 (0.088–1.000)	0.0088	Kruskal–Wallis H(3) = 11.62	0 (0–0) ᵃᵇ	2 (1–2) ᵇ	0 (0–0) ᵃ	0 (0–0) ᵃ	Nuclear pyknosis

Values are median (IQR). Lesion severity was scored on an ordinal scale (0 = none, 1 = mild, 2 = moderate, and 3 = severe). Omnibus testing used Kruskal–Wallis with Dunn post hoc tests and Bonferroni familywise control (within each lesion). Within rows, groups sharing a superscript letter do not differ (adjusted *p* ≥ 0.05). Effect size is ε^2^ for Kruskal–Wallis with percentile bootstrap 95% CI (5000 resamples; resampling within groups); because ε^2^ is bounded (0–1), percentile CIs may reach the upper bound (1.0).

## Data Availability

The data supporting the findings of this study are available within the article. Further details related to the study are available from the corresponding author upon reasonable request.

## Abbreviations

The following abbreviations are used in this manuscript:

ABC	Avidin–biotin–peroxidase complex
Al	Aluminium
AlCl3	Aluminium chloride
ANOVA	Analysis of variance
ARRIVE	Animal Research: Reporting of In Vivo Experiments
ATP	Adenosine triphosphate
AVMA	American Veterinary Medical Association
Bax	Bcl-2-associated X protein
Bcl-2	B-cell lymphoma 2
b.w.	Body weight
Caspase-3	Cysteine-aspartic protease-3
CI	Confidence interval
CPT1α	Carnitine palmitoyltransferase 1 alpha
DAB	3,3′-diaminobenzidine
GSH	Glutathione
GST	Glutathione S-transferase
H&E	Haematoxylin and eosin
HSP70	Heat shock protein 70
HSV	Hue, saturation, value
IL-1β	Interleukin-1 beta
IL-6	Interleukin-6
IL-18	Interleukin-18
IQR	Interquartile range
JNK	c-Jun N-terminal kinase
Ki-67	Kiel 67 antigen
LC	L-carnitine
NF-κB	Nuclear factor kappa B
NLRP3	NOD-like receptor family pyrin domain containing 3
OD	Optical density
PAS	Periodic acid–Schiff
PI3K	Phosphoinositide 3-kinase
PPAR-γ	Peroxisome proliferator-activated receptor gamma
RGB	Red, green, blue
ROI	Region of interest
ROS	Reactive oxygen species
SD	Standard deviation
Sirt1	Sirtuin 1
SOD	Superoxide dismutase
TGF-β1	Transforming growth factor beta 1
TIFF	Tagged image file format
TNF-α	Tumour necrosis factor alpha
